# Supplementary material for: All-optical closed-loop voltage clamp for precise control of muscles and neurons in live animals
Source: Nat Commun. 2023 Apr 6;14:1939. doi: 10.1038/s41467-023-37622-6 (PMC10079764; doi:10.1038/s41467-023-37622-6)
Supplement: Supplementary file 1 — Supplementary Information [file 41467_2023_37622_MOESM1_ESM.pdf]

All-optical closed-loop voltage clamp for precise control of muscles and neurons in live animals

Amelie C.F. Bergs[1,2], Jana F. Liewald[1,2], Silvia Rodriguez-Rozada[3], Qiang Liu[4, 5], Christin Wirt[1,2], Artur Bessel[6], Nadja Zeitzschel[1,2], Hilal Durmaz[1,2], Adrianna Nozownik[3], Holger Dill[1,2], Maëlle Jospin[7] Johannes Vierock[8], Cornelia I. Bargmann[4,9], Peter Hegemann[8], J. Simon Wiegert[3,10], Alexander Gottschalk[1,2,*]

Affiliations:

[1] Buchmann Institute for Molecular Life Sciences, Goethe University, Max-von-Laue-Strasse 15, D-60438 Frankfurt, Germany.

[2] Institute of Biophysical Chemistry, Goethe University, Max-von-Laue-Strasse 9, D-60438 Frankfurt, Germany.

[3] Research Group Synaptic Wiring and Information Processing, Center for Molecular Neurobiology Hamburg, University Medical Center Hamburg-Eppendorf, D-20251 Hamburg, Germany.

[4] Lulu and Anthony Wang Laboratory of Neural Circuits and Behavior, The Rockefeller University, New York, NY 10065, USA.

[5] Department of Neuroscience, City University of Hong Kong, Tat Chee Avenue, Kowloon Tong, Hong Kong, China.

[6] independent researcher: Melatener Strasse 93, D-52074 Aachen, Germany.

[7] Université Claude Bernard Lyon 1, Institut NeuroMyoGène, 8 avenue Rockefeller, 69008 Lyon, France

[8] Institute for Biology, Experimental Biophysics, Humboldt University, D-10115 Berlin, Germany.

[9] Chan Zuckerberg Initiative, Palo Alto, USA.

[10] Medical Faculty Mannheim, University of Heidelberg, Ludolf-Krehl-Strasse 13-17, D-68167 Mannheim, Germany.

* Corresponding author. Email: a.gottschalk@em.uni-frankfurt.de

Supplementary data

Supplementary Figures 1-10

Supplementary Table 1

Supplementary References

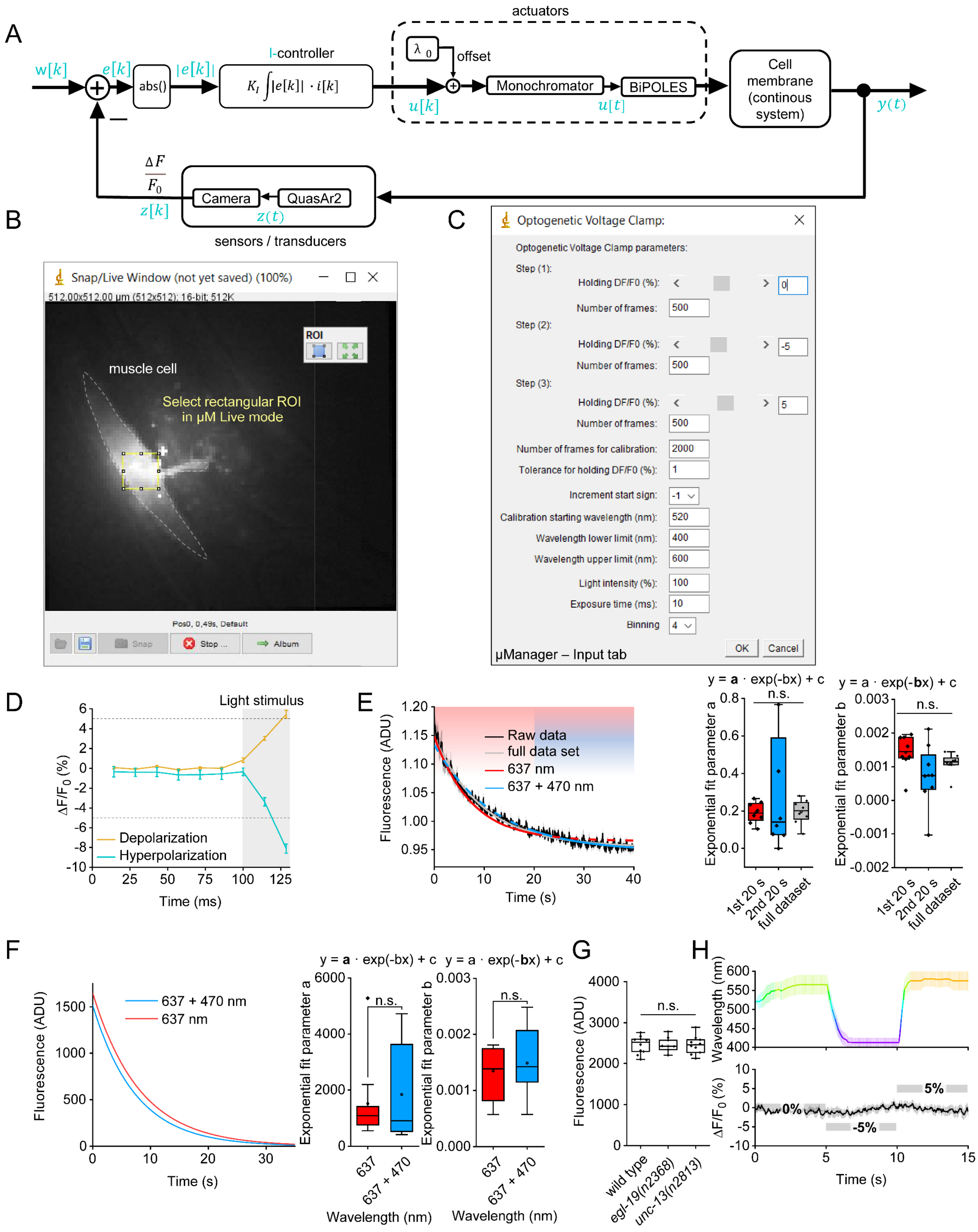

Supplementary Figure 1. Setup of the OVC software and control parameters. (A) Wiring diagram of I-controller: I-controller generates a digital control value u[k] based on error e[k] for the monochromator, which in turn generates analog light output u[t]. BiPOLES modulates membrane voltage y(t) upon analog light activation that in turn defines QuasAr2's fluorescence z(t) (transduced analog actual value). Fluorescence is monitored via camera and $\Delta F/F_0$ is

calculated (digital actual value). w[k]: Set point. i[k]: current sign of control error e[k]. **(B)** Rectangular ROI selection in µManager "live mode" prior to OVC measurement. **(C)** Input tab of the standard OVC software, implemented in µManager. **(D)** Step response analysis of the open loop system. System was challenged with de- and hyperpolarizing pulses (n=5 and n=8 independent animals, at 580 and 430 nm) to reach desired ±5% $\Delta F/F_0$ (shown is mean ± S.E.M.) and to approximate system time constant τ. **(E)** Left: Bleaching behavior and exponential fits of the fluorescence of QuasAr2 in BWMs, in control animals expressing no actuators (mean ± S.E.M.). Animals were illuminated with the 637 nm laser for the entire duration, and in addition, with 470 nm (300 µW/mm^2) for the last 20 s (n = 8 independent animals). Fits were performed for the first (red) or second 20 sec period (blue), or for the entire duration (grey). Middle and right panel: Statistical analysis of the deduced fit parameters. Parameter a: 1st 20 s / 2nd 20 s: P = 0.29777; 1st 20 s / full data set: P = 0.97414; 2nd 20 s / full data set: P = 0.63254; Parameter B: 1st 20 s / 2nd 20 s: P = 0.09717; 1st 20 s / full data set: P = 1; 2nd 20 s / full data set: P = 0.86977. **(F)** Exponential fits of QuasAr2 fluorescence as in (E) but illuminated with either 637 nm laser (n = 11 independent animals) or 637 nm laser and 470 (300 µW/mm^2) for the entire duration (n = 10 independent animals). Middle and right panels: Statistical analysis of the fit parameters of data in left panel. Parameter a: 637 + 470 / 637: P = 0.62431; Parameter b: 637 + 470 / 637: P = 0.56032. **(G)** Expression levels were assessed in wild type animals, and two different mutant strains expressing the OVC components (all from the same integrated transgene, *zxIs139*), using mean QuasAr fluorescence of comparable ROIs (n = 10, 9, 10 independent animals of the indicated genotypes, wild type, *egl-19(n2368)* and *unc-13(n2813)*). Coefficients of variation were 0.08 (wild type), 0.07 (*egl-19*) and 0.09 (*unc-13*). P values: *egl-19(n2368)* / wild type: 1; *unc-13(n2813)* / wild type: 1; *unc-13(n2813)* / *egl-19(n2368)*: 1. **(H)** Mean ± S.E.M. data for animals expressing only QuasAr2 in BWMs, while the OVC attempts to run a 0, -5, 5 % $\Delta F/F_0$ protocol (n = 8 independent animals). Upper panel: Monochromator wavelength; lower panel: mean fluorescence traces. Statistically significant differences analyzed by One-way ANOVA (in E, G) or two-sided t-test (in F), each with Bonferroni correction. Box plots (median, 25-75th quartiles); open dot: mean; whiskers: 1.5x inter-quartile range (IQR). Source data are provided as a Source Data file.

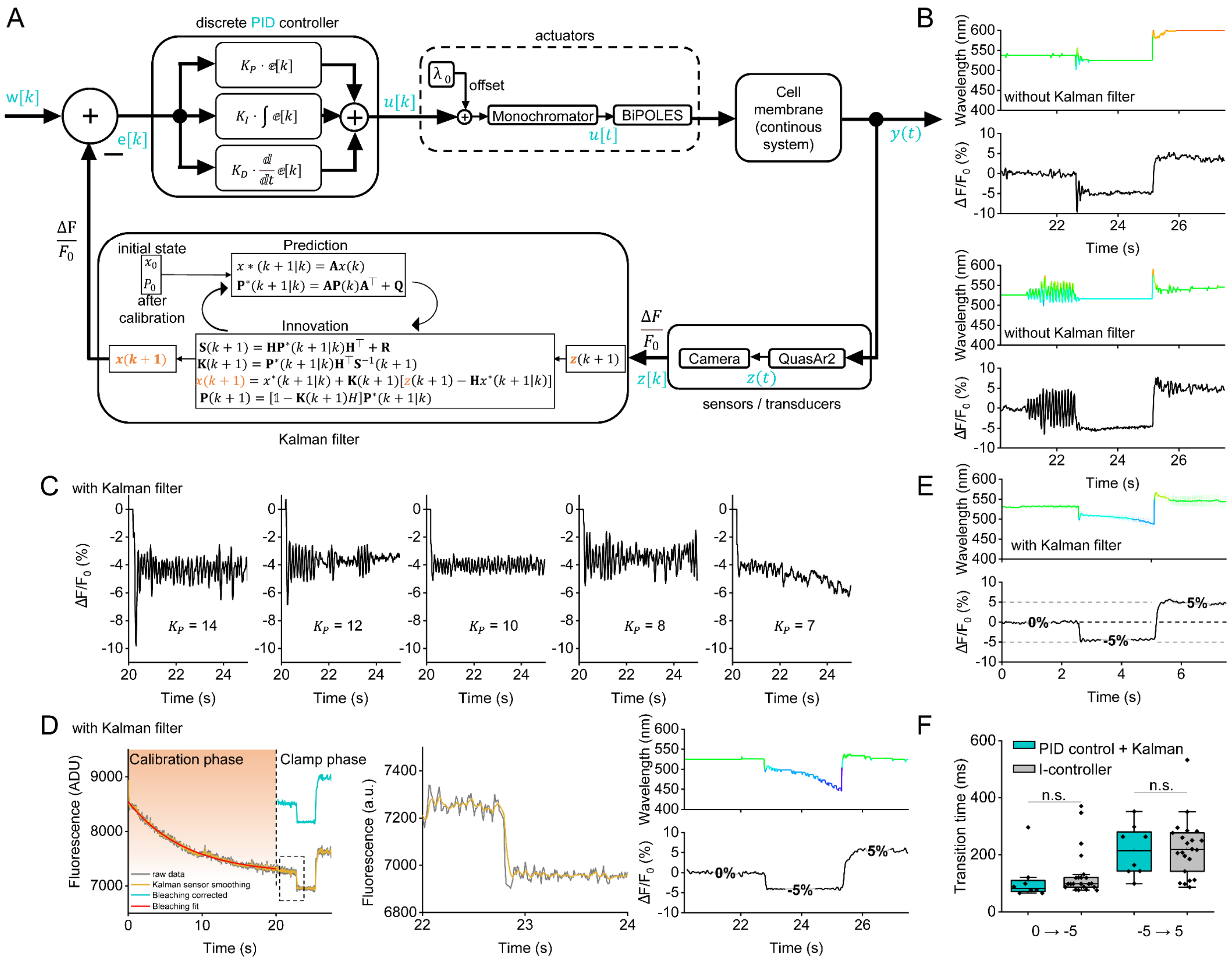

Supplementary Figure 2. Implementation of a PID controller with Kalman filter. (A) Wiring diagram of PID-controller with Kalman filter. Signal flow as in **Supplementary Fig. 1A** with the addition of a Kalman filter. **(B)** Comparison of single OVC experiments with PID control but without Kalman filter. Top: No oscillations. Bottom: With strong oscillations, despite identical parameters, emphasizing the need for a Kalman filter to obtain a stable PID-controller. **(C)** Ziegler-Nichols parameter tuning, with Kalman filter. **(D)** OVC three-step protocol (0, -5 and 5 % $\Delta F/F_0$) in BWMs, insets for close-up. Wavelength shown in the respective color, holding values are indicated for each step, yellow trace represents data processed with Kalman filter for sensor smoothing. Orange shade in left panel: transition period to reach tolerance range. **(E)** Upper panel: Overlay of mean (± S.E.M.) wavelength and (lower panel) fluorescence traces (n=8 independent animals; holding values: 0, -5, 5 % $\Delta F/F_0$). **(F)** Times required for the indicated 5 and 10 % $\Delta F/F_0$ transitions for PID- (plus Kalman filter) and I-controller (n = 8, 22, 8, 22 independent animals for these transitions, respectively: PID control + Kalman, 0 → -5; I-controller, 0 → -5; PID control + Kalman, -5 → 5; I-controller, -5 → 5). Statistically significant differences analyzed by Two-sided t-test with Bonferroni correction. Comparison 0 → -5, P = 0.52321; Comparison -5 → 5, P = 0.85972. Box plots (median, 25-75th quartiles); open dot: mean; whiskers: 1.5x inter-quartile range (IQR). Source data are provided as a Source Data file.

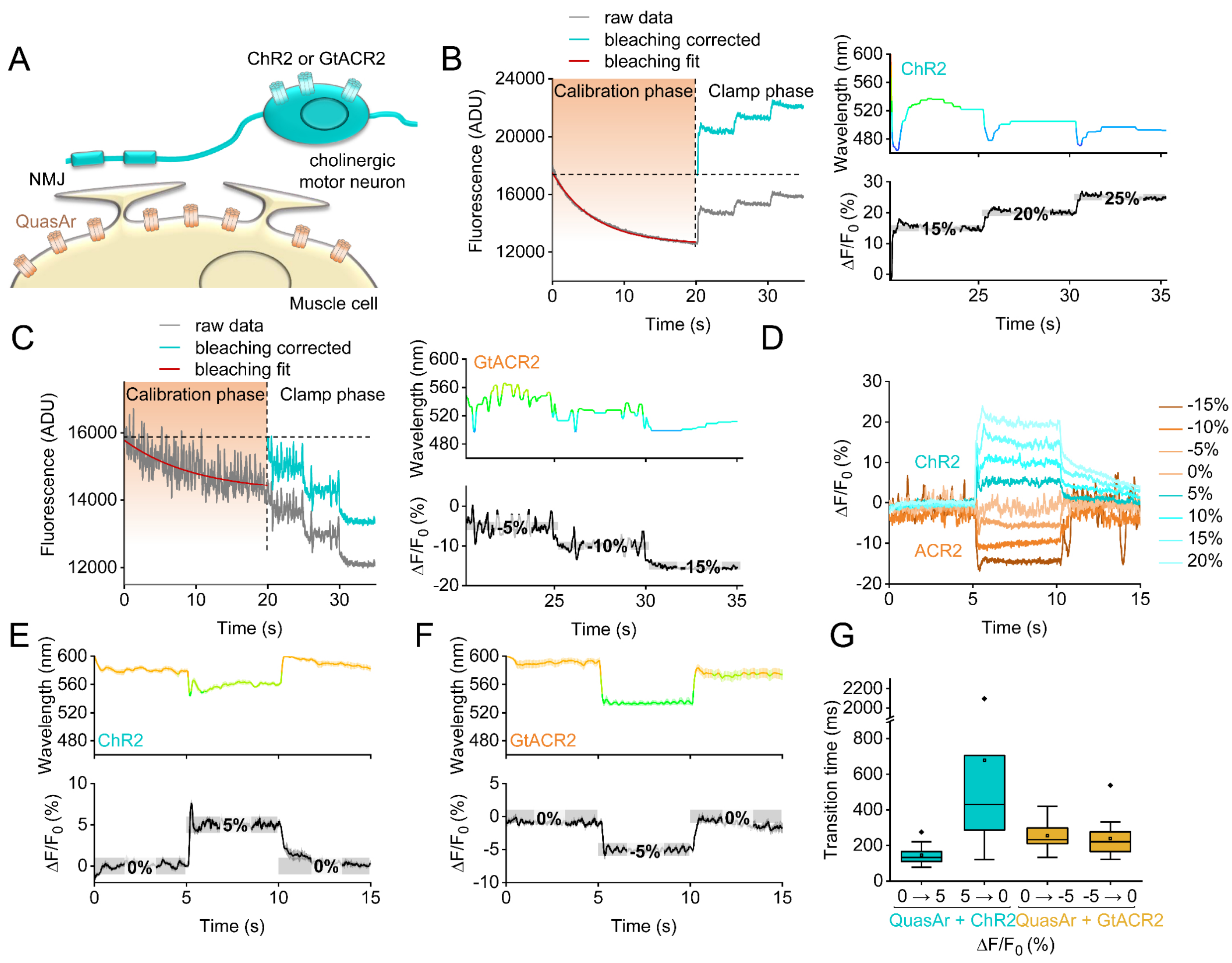

Supplementary Figure 3. Unidirectional steering of membrane voltage. (A) Cholinergic neurons express either ChR2 or *Gt*ACR2, while QuasAr2 is expressed in BWMs. **(B)** Three-step protocol in ChR2 animals. Left: Raw and bleaching corrected data of calibration (red: exponential fit) and clamping phase. Right panels: Corresponding wavelength and $\Delta F/F_0$ traces. Holding values 15, 20 and 25 % $\Delta F/F_0$; grey shade: tolerance range for each step. **(C)** Three-step (-5, -10 and -15 % $\Delta F/F_0$) protocol in *Gt*ACR2 animals, as in (B). **(D)** Single traces for both strains, 0 %, then -15 to 20 % $\Delta F/F_0$ (in 5% increments), and return to baseline. **(E)** Mean (± S.E.M.) traces (n = 17 independent animals); holding values: 0, 5, 0 % $\Delta F/F_0$, in ChR2 animals. **(F)** As in E, for *Gt*ACR2 animals (n = 14 independent animals; 0, -5, 0 % $\Delta F/F_0$). **(G)** Transition time, respective 5 % $\Delta F/F_0$ steps (n = 17, 17, 14, 13 independent animals for these transitions, respectively: 0 → 5, QuasAr+ChR2; 5 → 0; 0 → -5, QuasAr+ACR2; -5 → 0); box plots (median, 25-75th quartiles); open dot: mean; whiskers: 1.5x inter-quartile range (IQR). Source Data are provided as a Source Data file.

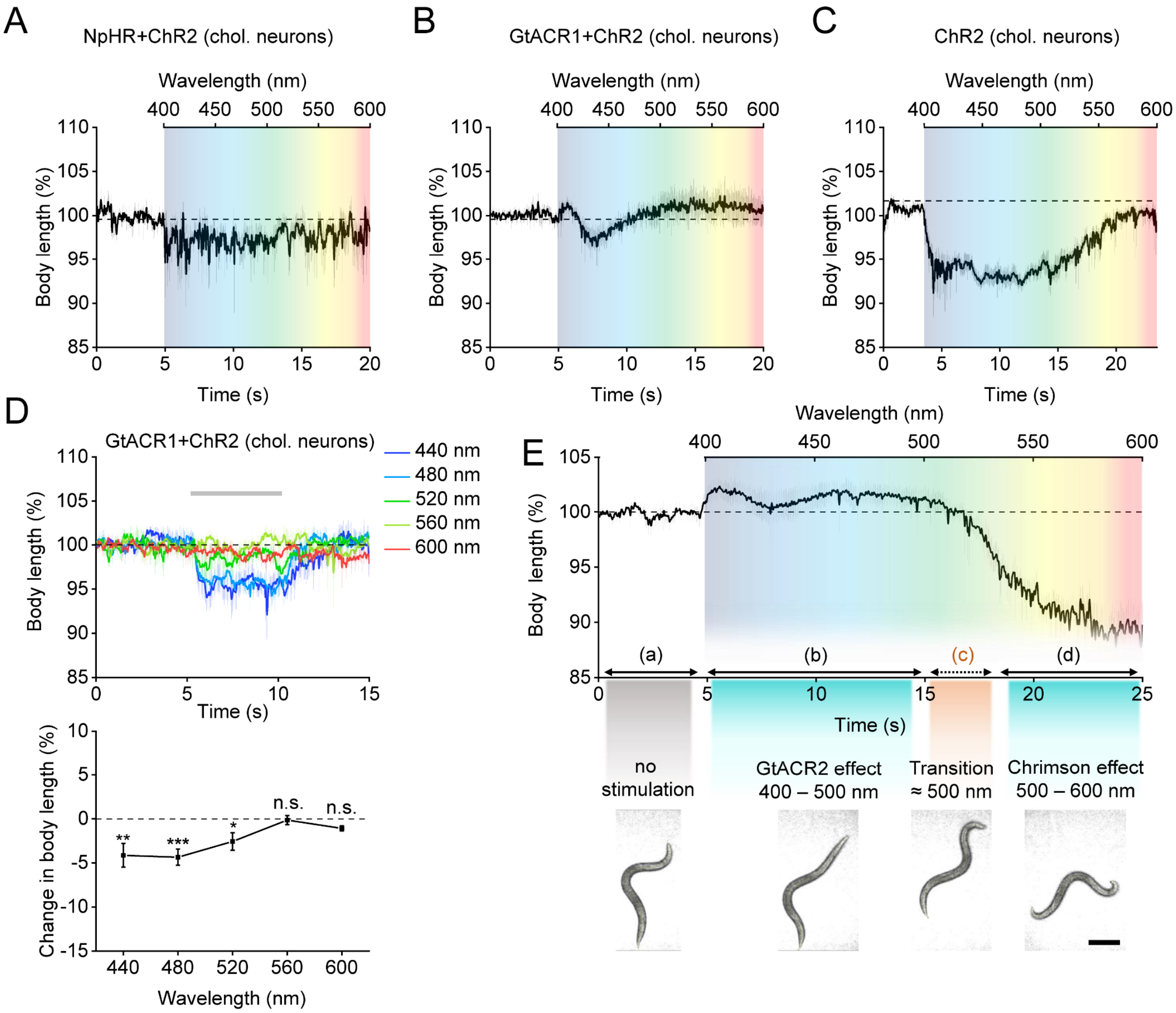

Supplementary Figure 4. Testing different combinations of depolarizing and hyperpolarizing actuators. (A-E) Body length measurements (mean ± S.E.M.) to test functionality of optogenetic actuator combinations, expressed in cholinergic neurons. **(A)** NpHR and ChR2 (n = 8 independent animals; monochromator wavelength ramp from 400 to 600 nm, 300 µW/mm^2). **(B)** *Gt*ACR1 and ChR2 (n = 10 independent animals). **(C)** ChR2-only (n = 8 independent animals). **(D)** *Gt*ACR1 and ChR2. Upper panel: 5 s light pulses, wavelength as indicated, 300 µW/mm^2 (n = 7, 7, 5, 6, 6 independent animals for the following wavelengths, respectively: 440, 480, 520, 560, and 600 nm). Lower panel: Mean (± S.E.M.) body length changes. Statistically significant differences were analyzed by one-way ANOVA with Bonferroni correction (P = 0,008577953; 0,000166674; 0,028628144; 0,476848228; 0,189584323 for the following wavelengths, respectively: 440, 480, 520, 560, and 600 nm). **(E)** Upper panel: Body length measurements of animals expressing BiPOLES in cholinergic neurons (mean ± S.E.M., wavelength ramp 400 - 600 nm, n = 8 independent animals). Below: Representative still images of animals for each phase of the experiment: (a) before light, (b) *Gt*ACR2 effect / muscle relaxation, (c) transition from hyper- to depolarization, (d) Chrimson effect / muscle contraction (scale bar: 200 µm). Source data are provided as a Source Data file.

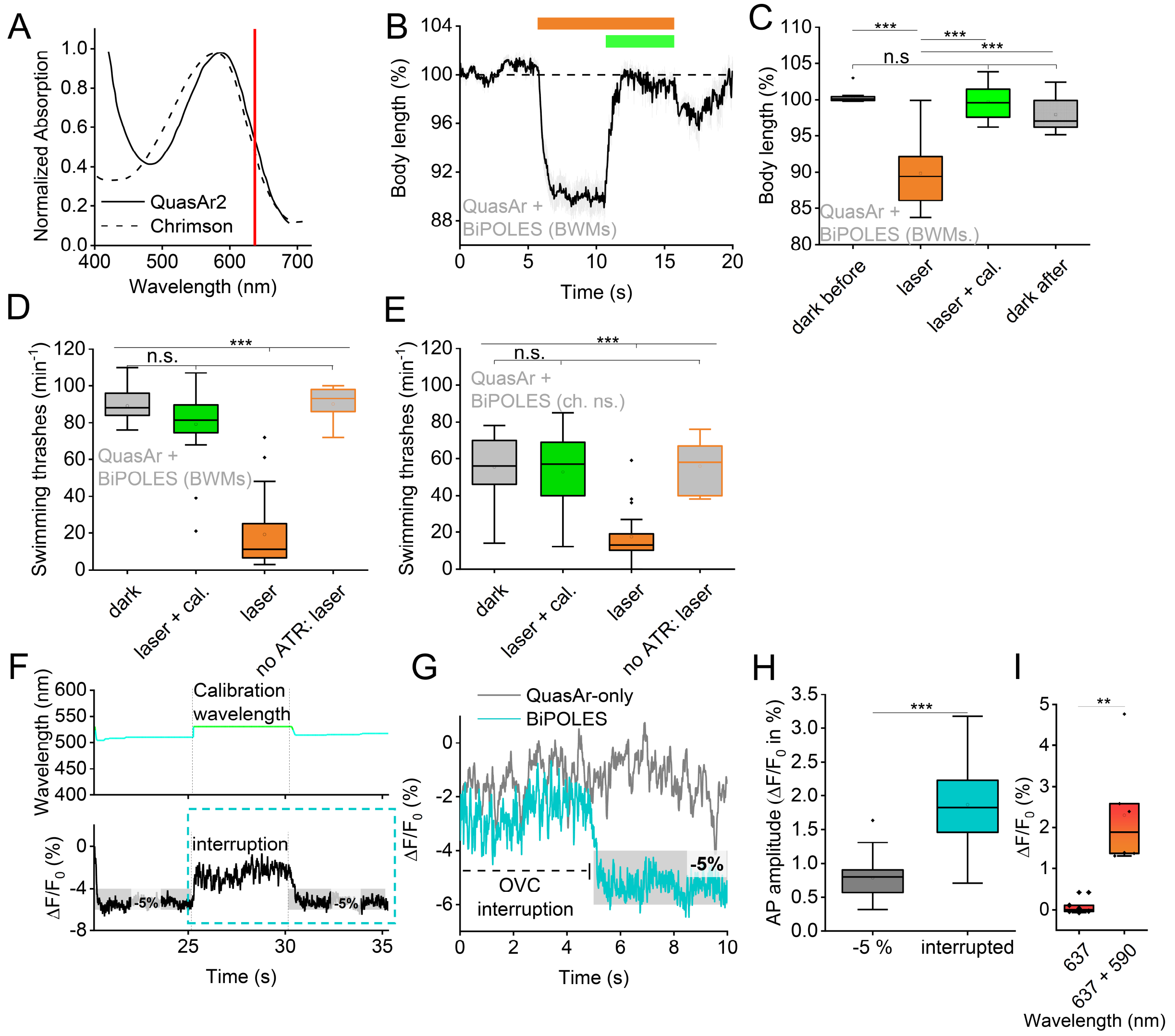

Supplementary Figure 5. BiPOLES activation by 637 nm laser light and calibration wavelength have no adverse effects on muscle function and locomotion. (A) Normalized absorption spectra of Chrimson and QuasAr2[1, 2], laser wavelength used for QuasAr2 imaging is indicated by a red line. **(B)** Body length measurements of animals expressing BiPOLES and Quasar in BWMs (mean ± S.E.M., light pulses: 637 nm laser and 637 nm laser plus calibration wavelength (ca. 521 nm, n = 14 independent animals). **(C)** Statistical analysis of data in (B). laser / dark before: p = 5.94E-13; laser + cal. / dark before: p = 1; laser + cal. / laser: p = 4.41E-12; dark after / dark before: p = 0.156; dark after / laser: p = 2.11E-09; dark after / laser + cal.: p = 0.55. **(D, E)** Analysis of swimming activity of animals expressing BiPOLES and Quasar in BWMs (D, n = 17, 19, 19, 8 independent animals for these conditions: dark, laser + cal., laser, no ATR: laser; statistical significance was: laser / dark: p = 3.16E-19; laser / laser+cal.: p = 3.36E-16; laser / no ATR: laser: p = 1.23E-14; dark / laser+cal.: p = 1; dark / no ATR: laser: p = 1; laser+cal. / no ATR: laser: p = 1) or cholinergic neurons (E, n = 17, 17, 17, 10 independent animals for these conditions: dark, laser + cal., laser, no ATR: laser; statistical significance was: laser / dark: p = 3.82E-07; laser / laser+cal.: p = 2.1E-06; laser / no ATR: laser: p = 7.57E-06; dark / laser+cal.: p = 1; dark / no ATR: laser: p = 1; laser+cal. / no ATR: laser: p = 1). Illumination parameters as in (C), or in the absence of all-*trans* retinal (ATR). **(F)** OVC experiment in BWMs (BiPOLES and QuasAr2) with clamp interruption (protocol: 1) holding value - 5 %, wavelength determined by OVC, 2) interruption at calibration wavelength, 3) holding value - 5 % $\Delta F/F_0$). **(G)** Close-up of (F, lower panel), with comparison to voltage fluorescence activity of

unstimulated animals expressing only QuasAr2. **(H)** Group data of $\Delta F/F_0$ fluorescence amplitudes of typical, action potential-based fluctuations during -5 % clamping, or during no OVC action (n = 30 independent animals, p=2.69E-13). Box plots (median, 25-75th quartiles); open dot: mean; whiskers: 1.5x inter-quartile range (IQR). **(I)** Mean $\Delta F/F_0$ QuasAr2 signal (n = 6 independent animals, p=0.0023) in BWMs (co-expressed with BiPOLES) in the presence of laser light (637 nm) and additional signal in response to Chrimson excitation light (590 nm). In (C-E, H, I): Statistically significant differences were analyzed by two-sided t-test or One-way ANOVA with Bonferroni correction (***P ≤ 0.001, **P ≤ 0.01, *P ≤ 0.05, and see above for exact values). Box plots (median, 25-75th quartiles); open dot: mean; whiskers: 1.5x inter-quartile range (IQR). Source data are provided as a Source Data file.

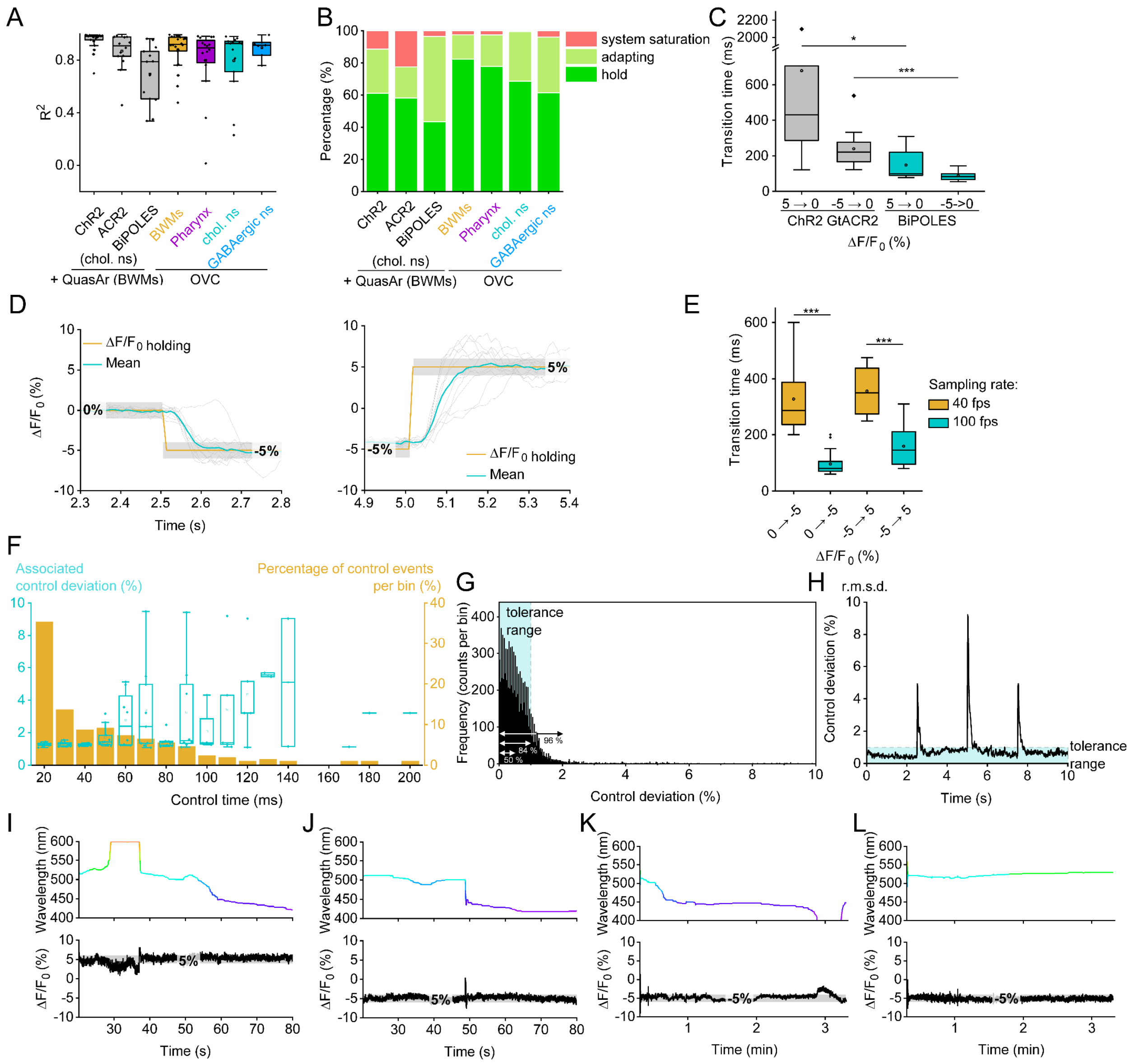

Supplementary Figure 6. Performance assessment, and long-term action of the OVC. (A) Quality of the exponential fit for bleach correction (coefficient of determination, R^2), for the different QuasAr2 / optogenetic actuator combinations, as indicated (n = 17, 14, 13, 22, 16, 14, 7 independent animals of these strains expressing: ChR2, ACR2, BiPOLES, OVC BWMs, OVC Pharynx, OVC chol. ns, OVC GABAergic ns). **(B)** Percentages of the clamping status (hold, adapting, system saturation), for the different QuasAr2 / optogenetic actuator combinations, as indicated (n = 17, 14, 13, 22, 16, 14, 7 independent animals of these strains expressing: ChR2, ACR2, BiPOLES, OVC BWMs, OVC Pharynx, OVC chol. ns, OVC GABAergic ns). **(C)** Summary of OVC speed, reflected by the time needed for a 5 % $\Delta F/F_0$ step for different configurations (single tools, same-cell approach; n = 17, 13, 10, 22 independent animals of these strains and conditions: ChR2, 5 → 0; ACR2, -5 → 0; BiPOLES, 5 → 0; BiPOLES, -5 → 0; statistically significant differences were: comparison: 5 → 0: ChR2 / BiPOLES: p = 0.01229; comparison: -5 → 0: ACR2 / BiPOLES: p = 3.8E-07). **(D)** Magnification of the transitions (left panel: 0 to -5 %, and right panel: -5 to 5% $\Delta F/F_0$) of the 4-step OVC protocol. Single traces are depicted in light grey, mean trace in cyan and $\Delta F/F_0$ holding in orange. **(E)** Comparison of transition times at 40 Hz and 100 Hz sampling rates (n = 8, 24, 8, 24 independent animals; 0 → -5 %: p = 1.38E-8; -5 → 5 %: p

= 4.13E-7). **(F)** Control events as a function of their duration and respective control deviations, measured in n = 11 independent animals. Orange bar graphs (right y-axis) represent the relative proportion of control events per control duration bin. The blue boxes (left y-axis) assign the respective control deviation that had to be overcome depending on the required control duration. Paired t test, P = 0.2271. **(G)** Histogram representation of all control deviations. Tolerance range is highlighted in blue. **(H)** Root-mean-square deviation of $\Delta F/F_0 - \Delta F/F_{0\ holding\ /\ target}$. Tolerance range is highlighted in blue. **(I-L)** Long-term OVC action, de- and hyperpolarizing steps. Shown are the achieved fluorescence values (lower panels, grey shades are tolerance range of the OVC protocol), and monochromator wavelength required (upper panels). Box plots (median, 25-75th quartiles); open dot: mean; whiskers: 1.5x inter-quartile range (IQR). Statistically significant differences were analyzed (C) by one-way ANOVA with Bonferroni correction (***P ≤ 0.001, *P ≤ 0.05). Source data are provided as a Source Data file.

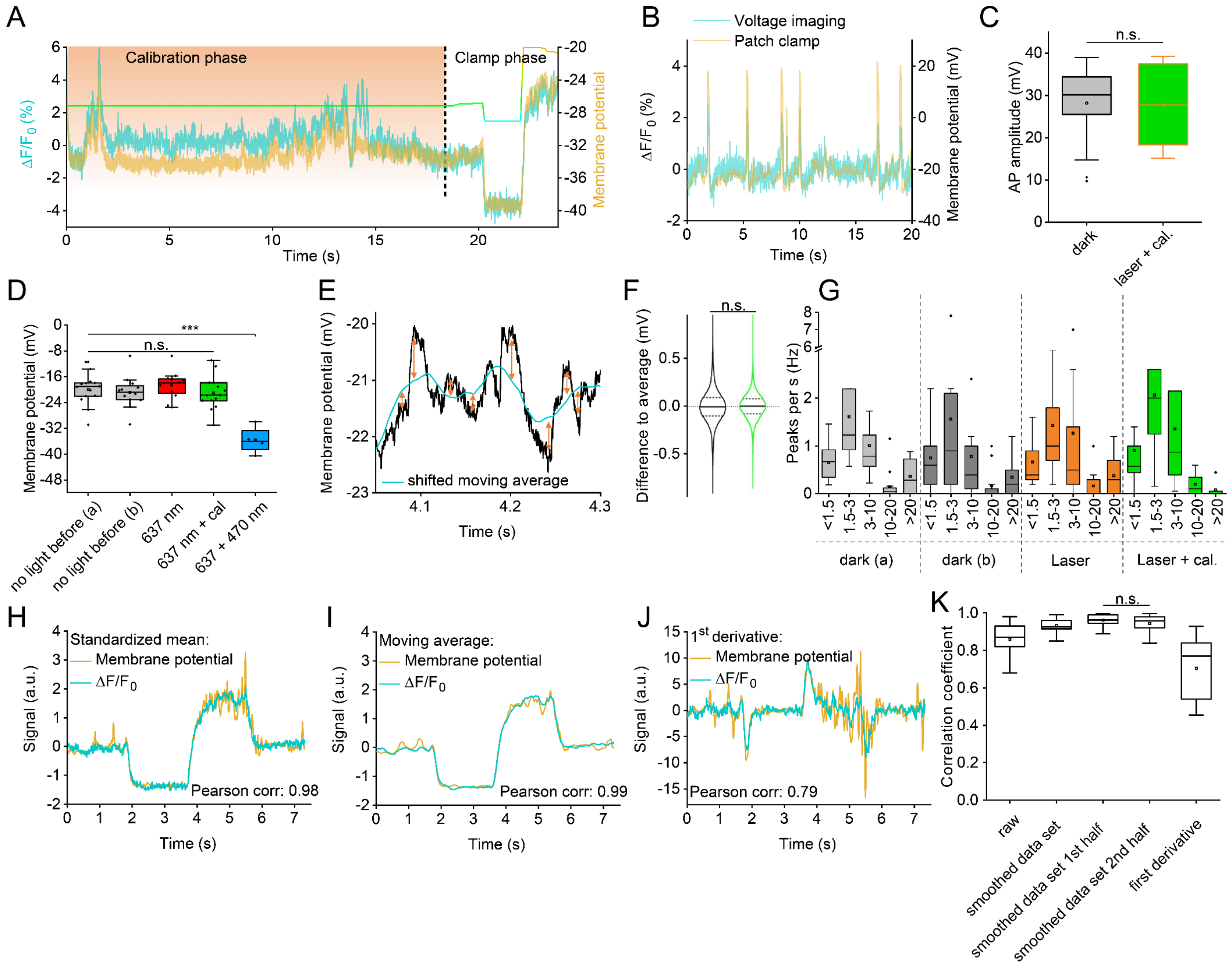

Supplementary Figure 7. Simultaneous patch-clamp and fluorescence measurement, normal membrane voltage behavior in BiPOLES-activated BWMs, no progressive error following calibration phase. (A) Original record of simultaneous voltage and fluorescence measurement during calibration and clamp phase (3 step OVC protocol, -3, +3 % $\Delta F/F_0$). Note the fluorescence trace was subsequently bleaching-corrected for the calibration phase. **(B)** APs in simultaneous patch-clamp and fluorescence recordings during OVC calibration phase (637 nm laser and 521 nm calibration wavelength). **(C)** Statistical analysis of AP amplitude. n = 4 independent animals, 24-27 APs. **(D)** Membrane potential in muscle was measured by patch-clamp under the indicated light conditions in n = 14, 12, 12, 14, 4 independent animals under these conditions: no light before (a), no light before (b), 637 nm, 637 nm + cal, 637 + 470 nm. **(E, F)** Analysis of small, subthreshold voltage fluctuations observed during patch-clamp, without or with 637 nm laser and calibration wavelength. Differences (arrows) of actual peaks to shifted moving average (blue), as a proxy for base line (E), were statistically analyzed in (F). n = 14 independent animals, with ca. 55.000 single data points per measurement. **(G)** Frequency distribution of distinct voltage signals, incl. APs, observed during patch-clamp recordings. Mean number of events (±S.E.M.) per second is shown as a function of peak amplitudes (in mV), for the different illumination conditions, as indicated below. n = 14 independent animals, 3118 peaks. None of the respective amplitude distributions showed significant differences to any other bin. **(H)** Patch-clamp derived membrane voltages (mean, yellow line) were compared to the mean $\Delta F/F_0$ levels (blue line) induced by the OVC during the clamp phase, based on parameters derived in the calibration phase (not shown). **(I)** As in (H), but mean moving averages were analyzed. **(J)** As in (H), but the signal change (1st derivative) was compared. **(K)** Statistical analyses of correlation coefficients

determined from data in (H-J; n = 14 independent animals), comparing the first and second halves of the experiments (p = 0.31). Correlation coefficients show high fidelity, typically >0.8. Statistically significant differences (***$P \leq 0.001$) were analyzed by one-way ANOVA with Bonferroni correction (in D, F, G, K) or paired, two-sided t-test (in C). Box plots (median, 25-75th quartiles); open dot: mean; whiskers: 1.5x inter-quartile range (IQR). Source data are provided as a Source Data file.

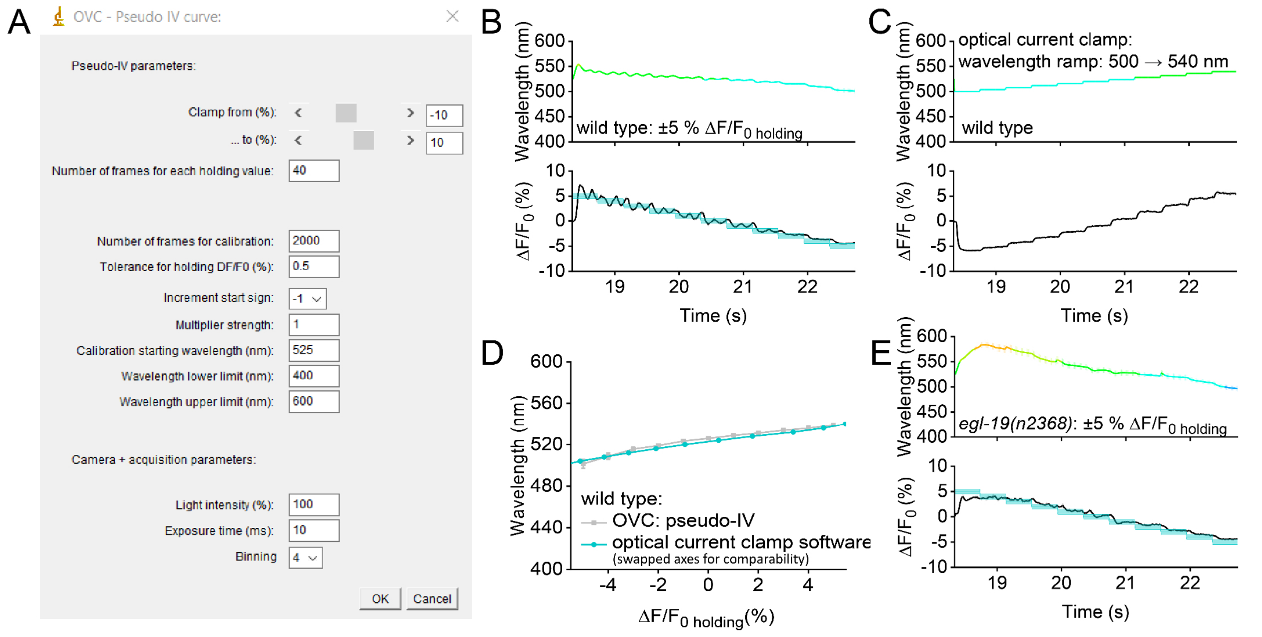

Supplementary Figure 8. Optical pseudo-I/V curve measurements: (A) Input tab of the "pseudo I/V curve" software allowing to run distinct % $\Delta F/F_0$ as clamp values (equivalent to voltages), while recording wavelengths (equivalent to currents). **(B)** Ramping +5 to -5 % $\Delta F/F_0$ (lower panel, mean ±S.E.M.; blue shades: tolerance ranges), while recording wavelengths (upper panel, mean ±S.E.M.; n = 17 independent animals). **(C)** Inverse experiment of (B), single trace, running a wavelength ramp (upper panel) and recording % $\Delta F/F_0$ (lower panel; n = 15 independent animals). **(D)** Comparison of OVC-based pseudo-I/V curve and measurement with the optical current clamp software (see **Supplementary Fig. 9A-C**), demonstrating high fidelity of the OVC control capabilities. **(E)** Measuring optical pseudo-I/V curves for *egl-19(n2368)* mutants, compare to (B) for wild type animals (n = 14 independent animals). Source data are provided as a Source Data file.

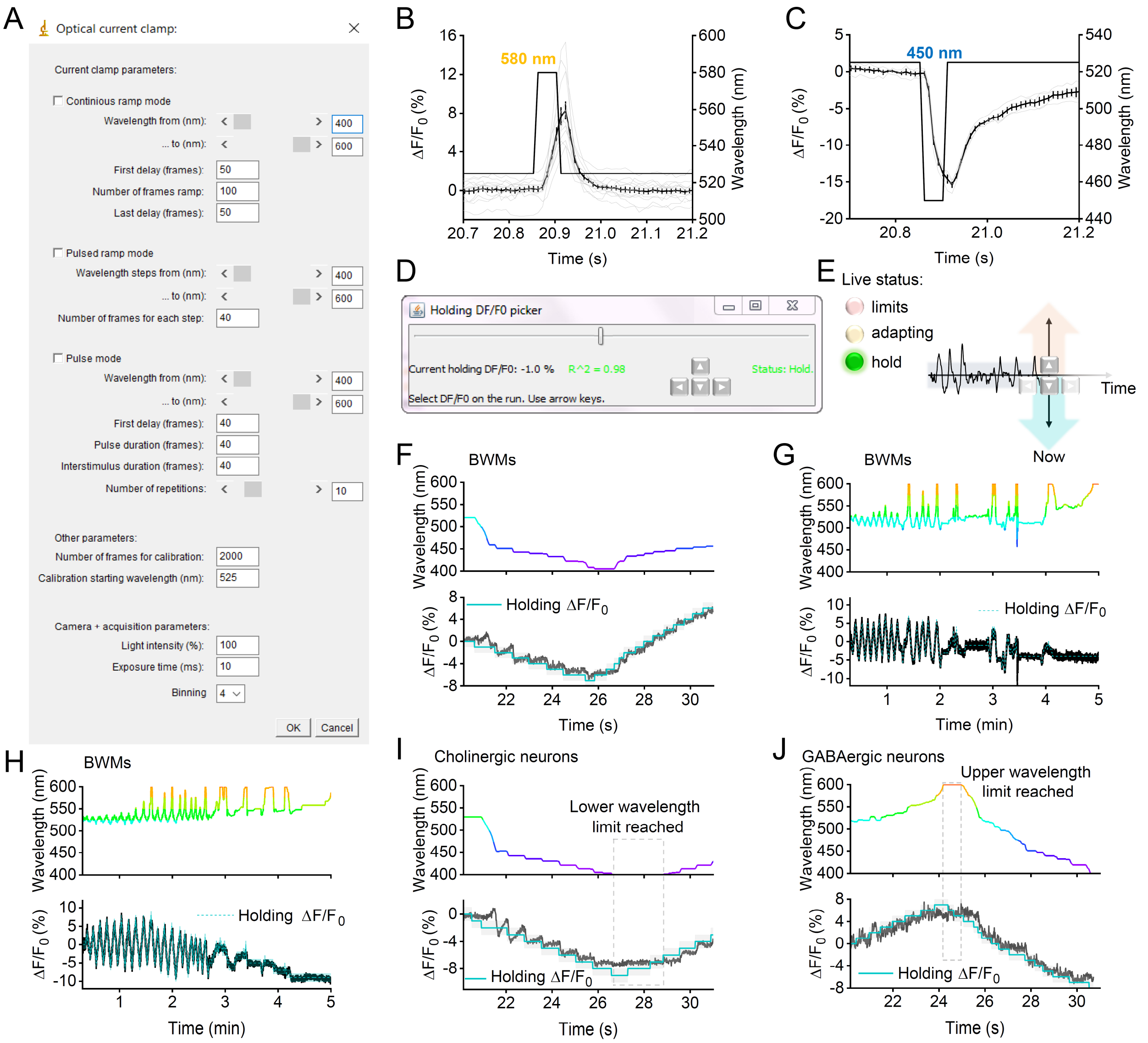

Supplementary Figure 9. Software for 'optical current clamp', and software for 'on-the-run' live voltage adjustment: (A) Software to achieve bidirectional optical current clamping, input tab. **(B, C)** Mean ±S.E.M. % $\Delta F/F_0$, resulting from 100 ms depolarizing (590 nm) step (B, n = 13 independent animals) or from a hyperpolarizing (450 nm) step (C, n = 5 independent animals). **(D-J)** Time-varying OVC live control ('on-the-run'). (D) User interface for software version allowing live control of membrane voltage fluorescence. (E) Scheme: Holding values can be selected using arrow keys. Live status (system on hold, adapting or exceeding limits) is shown, enabling adjustment. (F-H) Example traces of wavelength (upper panels) and holding values as % $\Delta F/F_0$ (lower panels) in BWMs for brief (F) and extended periods (G, H), as well as in cholinergic (I) and GABAergic neurons (J). Source data are provided as a Source Data file.

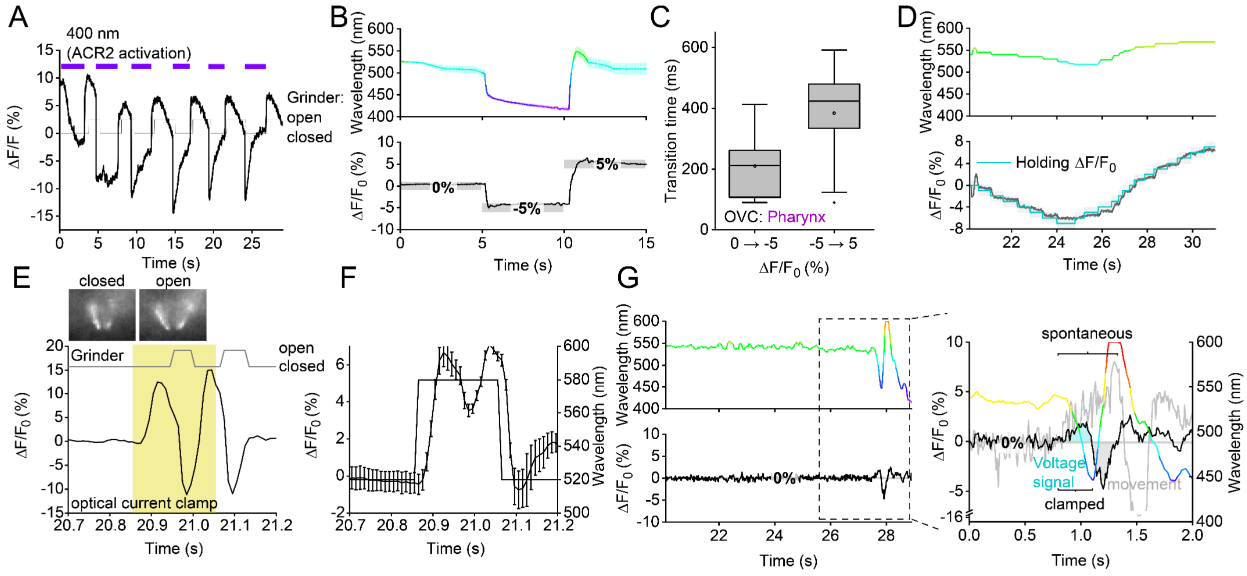

Supplementary Figure 10. Voltage imaging and OVC measurements in pharyngeal muscle and the motor neuron DVB. (A) Voltage imaging and analysis of grinder opening in animals expressing QuasAr2 and BiPOLES in pharyngeal muscle, stimulated with consecutive 400 nm light pulses (300 µW/mm^2). Corresponding open or closed state of the grinder as indicated. **(B)** Mean (± S.E.M.) traces (n = 16 independent animals) of OVC protocol in pharyngeal muscle (0, -5, 5 % $\Delta F/F_0$). **(C)** Transition time required by the OVC in the pharynx to execute 5 and 10 % $\Delta F/F_0$ steps (n = 16 independent animals). **(D)** "On-the-run"- experiment of OVC in pharyngeal muscle. **(E, F)** 'Optical current clamp' protocol, applied to pharyngeal muscle (yellow shade, depolarization evokes two APs), and assessing QuasAr fluorescence as a readout; single experiment (E), mean (± S.E.M.) fluorescence analysis (n = 9 independent animals, F). **(G)** Example DVB voltage fluorescence (lower panel) and wavelength (upper panel) traces upon suppression of an AP by the OVC. Inset: Close-up and overlay of spontaneous (light gray trace) and clamped (black trace) DVB voltage signal. Monochromator wavelength is presented in the respective color. Voltage signal and movement artefact highlighted in blue and grey, respectively. Box plots (median, 25-75th quartiles); open dot: mean; whiskers: 1.5x inter-quartile range (IQR). Source data are provided as a Source Data file.

Supplementary Table 1. Photon count and shot noise limited accuracy of the OVC system.

Conversion gain (e^-/count)		0.23	
QE at 700 nm		0.87	
Exposure time (s)		0.01	
Mean grey value per pixel (ADU)		19714 ± 1825	
		per pixel	full ROI
Signal	e^-	4534 ± 420	1578198 ± 176770
	photons	5212 ± 482	1814021 ± 203184
Photon shot noise (photons)		70 ± 3	1294 ± 69
Accuracy (%)		1.47 ± 0.05	0.08 ± 0.005

Source data are provided as a Source Data file.

Supplementary References

1. Oda, K. et al. Crystal structure of the red light-activated channelrhodopsin Chrimson. *Nature communications* **9**, 3949 (2018).
2. Zou, P. et al. Bright and fast multicoloured voltage reporters via electrochromic FRET. *Nature communications* **5**, 4625 (2014).
