# Supplementary material for: All-optical closed-loop voltage clamp for precise control of muscles and neurons in live animals
Source: Nat Commun. 2023 Apr 6;14:1939. doi: 10.1038/s41467-023-37622-6 (PMC10079764; doi:10.1038/s41467-023-37622-6)
Supplement: Supplementary file 3 — Description of Additional Supplementary Files [file 41467_2023_37622_MOESM3_ESM.pdf]

All-optical closed-loop voltage clamp for precise control of muscles and neurons in live animals

Bergs et al.

Description of Additional Supplementary Files

File Name: Supplementary Movie 1

Description: *C. elegans* expressing BiPOLES in cholinergic motor neurons, during a wavelength ramp from 400-600 nm. Animal crawling on solid substrate, with inactive (relaxed) muscle, transition to normal movement, followed by activated (contracted) muscle and respective paralysis, as indicated.

File Name: Supplementary Movie 2

Description: 'On-the-run' mode of the OVC, enabling adjusting membrane voltage during a running acquisition. Left panels, upper: monochromator wavelength, lower: voltage fluorescence trace. Arrow keys indicate the live settings chosen by the experimenter.

File Name: Supplementary Movie 3

Description: Dynamically clamping voltage in the pharynx, using the OVC. Video shows QuasAr fluorescence in the terminal bulb of the pharynx. Structures of the grinder of the pharynx are indicated (open and closed states), as well as the calibration and clamping phase. Overlaid are optical voltage traces (bottom), as well as the wavelength of the monochromator, used during clamping, as a color-coded trace at the top.

File Name: Supplementary Code 1

Description: Software scripts, to be implemented in MicroManager microscopy freeware, are provided as a zip archive, containing these scripts and documentation:

1) Main OVC software script
2) OVC ‚on-the-run' script
3) OVC four-step script
4) OVC pseudo-I/V script
5) Optical current clamp script
6) Software descriptions
