# Supplementary material for: All-optical closed-loop voltage clamp for precise control of muscles and neurons in live animals
Source: Nat Commun. 2023 Apr 6;14:1939. doi: 10.1038/s41467-023-37622-6 (PMC10079764; doi:10.1038/s41467-023-37622-6)
Supplement: Supplementary file 8 — Reporting Summary [file 41467_2023_37622_MOESM8_ESM.pdf]

nature portfolio

Corresponding author(s): Alexander Gottschalk

Last updated by author(s): Mar 8, 2023

Reporting Summary

Nature Portfolio wishes to improve the reproducibility of the work that we publish. This form provides structure for consistency and transparency in reporting. For further information on Nature Portfolio policies, see our Editorial Policies and the Editorial Policy Checklist.

Statistics

For all statistical analyses, confirm that the following items are present in the figure legend, table legend, main text, or Methods section.

n/a	Confirmed	
☐	☒	The exact sample size (n) for each experimental group/condition, given as a discrete number and unit of measurement
☐	☒	A statement on whether measurements were taken from distinct samples or whether the same sample was measured repeatedly
☐	☒	The statistical test(s) used AND whether they are one- or two-sided<br>*Only common tests should be described solely by name; describe more complex techniques in the Methods section.*
☒	☐	A description of all covariates tested
☐	☒	A description of any assumptions or corrections, such as tests of normality and adjustment for multiple comparisons
☐	☒	A full description of the statistical parameters including central tendency (e.g. means) or other basic estimates (e.g. regression coefficient) AND variation (e.g. standard deviation) or associated estimates of uncertainty (e.g. confidence intervals)
☐	☒	For null hypothesis testing, the test statistic (e.g. F, t, r) with confidence intervals, effect sizes, degrees of freedom and P value noted<br>*Give P values as exact values whenever suitable.*
☒	☐	For Bayesian analysis, information on the choice of priors and Markov chain Monte Carlo settings
☒	☐	For hierarchical and complex designs, identification of the appropriate level for tests and full reporting of outcomes
☐	☒	Estimates of effect sizes (e.g. Cohen's d, Pearson's r), indicating how they were calculated

Our web collection on statistics for biologists contains articles on many of the points above.

Software and code

Policy information about availability of computer code

Data collection	µManager 1.4.22, PatchMaster software 2.71 (Heka), SutterPatch V2 (Sutter Instruments); MATLAB 2016b, 2019b (Mathworks). Custom data collection scripts (Beanshell) for µManager are provided as supplementary data.
Data analysis	Data analysis was performed in Origin Pro 2021, Microsoft Excel 2016, 2019, SutterPatch V2 (Sutter Instrument); MATLAB 2016b, 2019b (Mathworks), or ImageJ (1.53c), with statistics calculated in Graph Pad Prism 8.02, Origin Pro 2021, MATLAB 2016b, 2019b (Mathworks), ImageJ (1.53c), or Stata 12 ic. Custom data analysis scripts (Beanshell) for µManager are provided as supplementary data. The decision tree algorithm that is part of the OVC main software, and all OVC software variants, are provided as supplementary information.

For manuscripts utilizing custom algorithms or software that are central to the research but not yet described in published literature, software must be made available to editors and reviewers. We strongly encourage code deposition in a community repository (e.g. GitHub). See the Nature Portfolio guidelines for submitting code & software for further information.

Data

Policy information about availability of data

All manuscripts must include a data availability statement. This statement should provide the following information, where applicable:

- Accession codes, unique identifiers, or web links for publicly available datasets
- A description of any restrictions on data availability
- For clinical datasets or third party data, please ensure that the statement adheres to our policy

The source data have been provided with the manuscript. The videos used to retrieve this raw data are available from the authors. They are small ROIs chosen from

the respective field of view, but typically represent a few percent of the total image. Their information content thus is not higher than the grey values listed for each time frame in the source data, provided for each figure. The electrophysiology recording data (data tables) is provided in the source data.

Human research participants

Policy information about studies involving human research participants and Sex and Gender in Research.

Reporting on sex and gender	*Use the terms sex (biological attribute) and gender (shaped by social and cultural circumstances) carefully in order to avoid confusing both terms. Indicate if findings apply to only one sex or gender; describe whether sex and gender were considered in study design whether sex and/or gender was determined based on self-reporting or assigned and methods used. Provide in the source data disaggregated sex and gender data where this information has been collected, and consent has been obtained for sharing of individual-level data; provide overall numbers in this Reporting Summary. Please state if this information has not been collected. Report sex- and gender-based analyses where performed, justify reasons for lack of sex- and gender-based analysis.*
Population characteristics	*Describe the covariate-relevant population characteristics of the human research participants (e.g. age, genotypic information, past and current diagnosis and treatment categories). If you filled out the behavioural & social sciences study design questions and have nothing to add here, write "See above."*
Recruitment	*Describe how participants were recruited. Outline any potential self-selection bias or other biases that may be present and how these are likely to impact results.*
Ethics oversight	*Identify the organization(s) that approved the study protocol.*

Note that full information on the approval of the study protocol must also be provided in the manuscript.

Field-specific reporting

Please select the one below that is the best fit for your research. If you are not sure, read the appropriate sections before making your selection.

[x] Life sciences [ ] Behavioural & social sciences [ ] Ecological, evolutionary & environmental sciences

For a reference copy of the document with all sections, see nature.com/documents/nr-reporting-summary-flat.pdf

Life sciences study design

All studies must disclose on these points even when the disclosure is negative.

Sample size	No statistical methods were applied to predetermine sample size as the effect size was not known before the study. However, sample sizes reported here for the different experiments were matched to published experiments that used similar methodology, model systems and manipulations (for C. elegans electrophysiology, see DOI: https://doi.org/10.1038/nmeth.1252; for voltage imaging, see: DOI: https://doi.org/10.1073/pnas.1902443116; for optogenetics experiments, see: DOI: 10.1038/nature05744 and DOI: https://doi.org/10.1038/nmeth.1252). For the experiments in mammalian neurons presented in this study, sample sizes were matched to published experiments that used similar methodology and manipulations: DOI: 10.1038/s42003-022-03636-x, DOI: 10.1038/s41467-021-24759-5, DOI: 10.1038/s41467-018-06421-9, DOI: 10.1038/s41598-017-14330-y
Data exclusions	Generally, no data were excluded during the analysis workflow. However, In patch-clamp experiments with hippocampal neurons, we excluded recordings that were not stable throughout the duration of the experiment or where a proper recording configuration could not be achieved. Recordings with a membrane resistance below 50 MOhm and a series resistance over 30 MOhm were not analyzed.
Replication	For experiments involving C. elegans, measurements were performed on at least three independent animal generations and compared to each other (with no significant changes unless noted), or were repeated until at least three successful replications were obtained (experiments were considered un-successful if technical problems prevented normal data acquisition, or if animals were too small, or appearedsick, e.g. due to bacterial contaminations on the present growth medium). These measurements were then pooled for analysis as a single group. For rodent data, all experiments were replicated two to four times in biologically independent samples (neurons in hippocampal slices). The precise n numbers are reported in the manuscript.
Randomization	Animals for imaging analysis were randomly selected from a given population of distinct genotype. Animals were picked the night before the experiment, as L4 larvae, thus ensuring similar age of the animals on the day of the experiment. Experiments were repeated with animals from different, independent cultures on different days. For C. elegans electrophysiological analysis, there is a bias towards larger animals due to the difficulty of dissection of small animals. Hippocampal slice cultures were randomly chosen for virus transduction and single-cell electroporation. Fluorescent transgene-expressing cells were randomly selected for patch-clamp and voltage-imaging experiments, provided they showed intact morphology in the DIC image.
Blinding	No blinding was required for C. elegans measurements (investigators were not blinded to group allocation during data collection), since analysis was performed by a consistent workflow, age-matched animals were collected the day beofre the experiment, and all single experiments were included in the analyses. Data analysis of hippocampal neuronal recordings was not done blinded, because opsin-mediated effects were apparent in the recordings and thereby revealed the condition. However, pre-established, semi-automatic stimulation protocols were used, which could not be influenced by the experimenter. Also pre-established analysis pipelines were used that did not permit dismissal

of any data points or experiments by the analyst based on the analysis result.

Reporting for specific materials, systems and methods

We require information from authors about some types of materials, experimental systems and methods used in many studies. Here, indicate whether each material, system or method listed is relevant to your study. If you are not sure if a list item applies to your research, read the appropriate section before selecting a response.

Materials & experimental systems

n/a	Involved in the study
☒	☐ Antibodies
☒	☐ Eukaryotic cell lines
☒	☐ Palaeontology and archaeology
☐	☒ Animals and other organisms
☒	☐ Clinical data
☒	☐ Dual use research of concern

Methods

n/a	Involved in the study
☒	☐ ChIP-seq
☒	☐ Flow cytometry
☒	☐ MRI-based neuroimaging

Animals and other research organisms

Policy information about studies involving animals; ARRIVE guidelines recommended for reporting animal research, and Sex and Gender in Research

Laboratory animals

Caenorhabditis elegans, Organotypic hippocampal slices were prepared from non-transgenic Wistar rats (Janvier labs) of both sexes at postnatal days 5-7.
C. elegans strains used:
ZX2476: zxEx1139[pmyo-3::QuasAr2; pmyo-2::CFP]
ZX2482: zxEx1145[pmyo-3::QuasAr2; pmyo-2::CFP]; zxIs5[punc-17::ChR2(H134R)::yfp;lin-15+]
ZX2483: zxEx1146[punc-17::ACR2::eYFP; pmyo-3::QuasAr2; pelt-2::GFP]
ZX2586: zxEx1228[punc-17::GtACR2::mCerulean::betaHK::Chrimson; pelt-2::GFP]
ZX2714: zxEx1250[punc-17::GtACR2::mCerulean::betaHK::Chrimson; pmyo-3::QuasAr2; pelt-2::GFP]
ZX2753: zxEx1266[pmyo-3::GtACR2::mCerulean::betaHK::Chrimson; pmyo-3::QuasAr2; pmyo-2::CFP]
ZX2755: zxEx1268[punc-47::QuasAr2::GFP; pmyo-2::CFP]
ZX2826: zxEx1282[pmyo-2::QuasAr2; pmyo-2::GtACR2::mCerulean::betaHK::Chrimson; pmyo-3::CFP]
ZX2827: zxEx1283[punc-17::GtACR2::mCerulean::betaHK::Chrimson; punc-17::QuasAr2; pelt-2::GFP]
ZX2828: zxEx1284[punc-47::QuasAr2::GFP; punc-47::GtACR2::mCerulean::betaHK:: Chrimson; pmyo-2::CFP]
ZX2876: zxIs139[pmyo-3::GtACR2::mCerulean::betaHK::Chrimson; pmyo-3::QuasAr2; pmyo-2::CFP]
ZX2935: unc-13(n2813); zxIs139[pmyo-3::GtACR2::mCerulean::betaHK::Chrimson; pmyo-3::QuasAr2; pmyo-2::CFP]
ZX3074: egl-19(n2368); zxIs139[pmyo-3::GtACR2::mCerulean::betaHK::Chrimson; pmyo-3::QuasAr2; pmyo-2::CFP].

Wild animals

This study did not involve wild animals.

Reporting on sex

We randomly used slice cultures from both sexes. For C. elegans, we only used hermaphrodites.

Field-collected samples

This study did not involve samples collected from the field.

Ethics oversight

Studies on C. elegans do not require ethics oversight. For rodent work, all procedures were performed in compliance with German law and in accordance with the guidelines of Directive 2010/63/EU. Protocols for slice culture experiments were performed under the protocol ORG997, which was approved by the local authorities ("Behörde für Justiz und Verbraucherschutz", City of Hamburg).

Note that full information on the approval of the study protocol must also be provided in the manuscript.
